# Intensified LOHC-Dehydrogenation Using Multi-Stage Microstructures and Pd-Based Membranes

**DOI:** 10.3390/membranes8040112

**Published:** 2018-11-19

**Authors:** Alexander Wunsch, Marijan Mohr, Peter Pfeifer

**Affiliations:** Institute for Micro Process Engineering, Karlsruhe Institute for Technology, 76344 Eggenstein-Leopoldshafen, Germany; alexander.wunsch@kit.edu (A.W.); Marijan.Mohr@gmx.de (M.M.)

**Keywords:** LOHC, dehydrogenation, multi-stage, PdAg-membrane, micro reactor, hydrogen purification

## Abstract

Liquid organic hydrogen carriers (LOHC) are able to store hydrogen stably and safely in liquid form. The carrier can be loaded or unloaded with hydrogen via catalytic reactions. However, the release reaction brings certain challenges. In addition to an enormous heat requirement, the released hydrogen is contaminated by traces of evaporated LOHC and by-products. Micro process engineering offers a promising approach to meet these challenges. In this paper, a micro-structured multi-stage reactor concept with an intermediate separation of hydrogen is presented for the application of perhydro-dibenzyltoluene dehydrogenation. Each reactor stage consists of a micro-structured radial flow reactor designed for multi-phase flow of LOHC and released hydrogen. The hydrogen is separated from the reactors’ gas phase effluent via PdAg-membranes, which are integrated into a micro-structured environment. Separate experiments were carried out to describe the kinetics of the reaction and the separation ability of the membrane. A model was developed, which was fed with these data to demonstrate the influence of intermediate separation on the efficiency of LOHC dehydrogenation.

## 1. Introduction

In the context of the energy transition, various technologies are investigated to store fluctuating renewable energy over periods of varying lengths. In contrast to batteries, electrical energy can be converted into chemical energy in the form of hydrogen by electrolysis. This hydrogen may serve as a particularly clean and climate-neutral energy source for mobility and stationary applications in the future. With approximately 33 kWh/kg, hydrogen has the highest mass related energy density of all fuels. However, storage is difficult because the substance is a gas under atmospheric conditions and has a low volumetric energy density. Common solutions such as compressed hydrogen at 350 or 700 bar (0.8 or 1.3 kWh_th_/L_H2_) and liquid hydrogen (2.4 kWh_th_/L_H2_) only provide partial benefits due to the high-risk potential and the difficult handling. An alternative technology is the storage of hydrogen in a so-called Liquid Organic Hydrogen Carrier (LOHC). The LOHC can be loaded with hydrogen (LOHC+) or unloaded (LOHC-) by reversible hydrogenation. The LOHC serves as a “deposit bottle” [1,2,3,4]. The continuous further development of the hydrogen network calls for a technology that pursues a decentralized approach. For example, micro-structured dehydrogenators with palladium membranes can be used in hydrogen filling stations, trains, or tankers to provide high-quality hydrogen from compact dehydrogenation units.

The perhydro-dibenzyltoluene (18H-DBT, LOHC+)/dibenzyltoluene (0H-DBT, LOHC-) system proves to be a promising LOHC since the aromatic compound can absorb up to nine molecules of hydrogen [5]. DBT is commercially used as a heat transfer fluid (e.g., Marlortherm SH from Sasol) with a great availability. In addition to a storage density of 57 g_H2_/L_18H-DBT_ (equivalent to 1.9kWh_H2_/L_18H-DBT_), the isomeric mixture is easy to store under atmospheric conditions due to its high stability. Furthermore, the material system is flame-resistant, neither explosive nor toxic, and is, therefore, not considered a hazardous good. This results in attractive opportunities to use the existing infrastructure via tank trucks for the distribution of the LOHC [6,7,8].

The loading/unloading of the LOHC can only take place under certain conditions (pressure, temperature) and in the presence of a catalyst. Thermodynamically favorable conditions for the release are temperatures between 280–330 °C and pressures below 5 bar. Under these conditions, the LOHC is dehydrogenated in the liquid state. During the reaction, a multiphase flow forms due to the released hydrogen. Due to the stoichiometry of 9 mol_H2_/mol_18H-DBT_, an enormous gas quantity forms even at low conversion rates in comparison to the liquid amount. As a result, part of the LOHC evaporates due to its non-negligible vapor pressure and is detectable in traces in the product gas despite subsequent condensation. Furthermore, low-boiling by-products are also found in the product gas. In order to supply hydrogen in high purity, e.g., for the operation of fuel cells, a purification step is necessary. Good heat management is also crucial for high catalyst and reactor related hydrogen productivity. Approximately 71 kJ/mol_H2_ heat is required for the release, which leads to a huge heat demand when the carrier is completely dehydrogenated (639 kJ/mol_18H-DBT_) [5]. 

**Figure 1:**
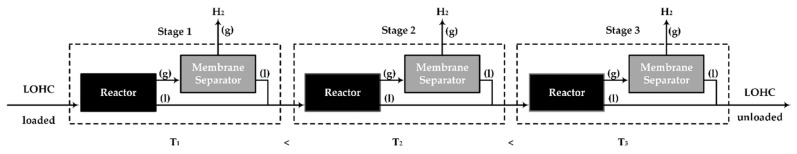
Schematic of the multi-stage approach with intermediate separation of hydrogen for the dehydrogenation of liquid organic hydrogen carriers. Most of the produced hydrogen is removed from the system after each reactor stage.

Micro-process engineering offers two promising approaches—the use of a micro-structured reactor allows an almost isothermal reaction environment, which prevents “cold spots” by limited heat input. Furthermore, it has already been shown that Pd-based membranes benefit from a micro-structured environment to separate hydrogen with high efficiency and purity from a gas mixture [9,10,11,12,13,14,15,16]. The combination of membrane and reaction in microreactors has been demonstrated as highly beneficial over conventional systems in these studies. Nevertheless, in the current reaction system, a fundamental difference exists, which is related to two major obstacles in the combination of membrane and micro-reactor. This includes the occurrence of liquid, which can prevent reasonable hydrogen permeation through the membrane, and the difficult phase contact between the liquid and the catalyst. A combination of the two process steps may be difficult but not inconceivable in previously proposed micro-reactor arrangements. In our opinion, the easiest approach to reasonable process intensification may be possible by a multi-stage dehydrogenation process with intermediate separation of hydrogen by Pd-based membranes (see Figure 1) with also an intelligent microchannel design fostering phase contacting presented in our current study. Overall, the multistage concept is attractive in two aspects. The separation removes most of the gas for the consecutive reactor stage in addition to its original function, which is the purification of hydrogen. Hydrogen removal minimizes the negative influence of the gas phase on the residence time of the liquid in the reactor part. Typical solutions to foster the conversion like an increase of the reaction temperature and higher residence times can cause catalyst deactivation by coke formation on the catalysts’ surface area. With the multi-stage approach, it may be possible to avoid the deactivation since, with an increasing number of reactor stages, less LOHC per stage must be converted and dry-out of the catalyst surface is less pronounced. A temperature stepping of the various reactors may further overcome the kinetic inhibition by increased formation of unloaded LOHC. However, the separation only works under a concentration gradient of hydrogen between the retentate and permeate side and, thus, the overall system pressure must be increased compared to the conventional process. This leads to decreasing equilibrium conversion.

An integrated system, not explored in this study, represents a virtually infinite number of separation stages, which, at first sight, is much more advantageous. However, the previously mentioned obstacle that the LOHC could at least partially wet the membrane surface area may lead to a drop in the separation efficiency. Furthermore, it is also unknown how strongly gas and liquid interact with each other in the microreactor and how much the residence times differ. Therefore, the multi-stage concept is ideally suited for investigating these phenomena in more detail.

According to our calculations with already applied membrane modules [16], considering the relatively low concentrations of other components in the gas phase of a separation at low reaction pressures (4–5 bar (a)) is feasible and the disadvantage of the shifted reaction equilibrium is, therefore, acceptable. In this work, the micro-structured multi-stage reactor concept with intermediate hydrogen separation (schematically shown in Figure 1) is modelled based on experimental data of the reactor and membrane separator.

## 2. Materials and Methods

### 2.1. Radial Flow Reactor

**Figure 2:**
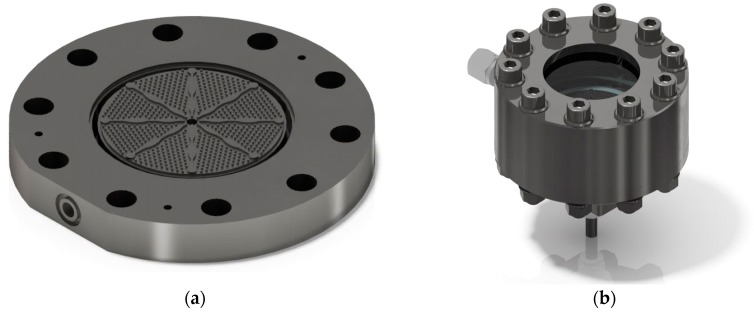
CAD schematic of the radial flow reactor: (**a**) The microstructure in detail including the space between the pins, which is filled with catalyst particles. The reactants are entering in the center and the reaction mixture flows from the inside to the outside and is removed in the form of a ring. (**b**) View of the reactor. A look inside is possible by using a glass plate pressed onto the structure.

A micro-structured radial flow reactor was developed for the challenging multiphase reaction. A CAD scheme of the microstructure can be seen in Figure 2a. The reactants enter at the center of the reaction chamber, flow out radially, and are collected in a ring. The microstructure is divided into eight equally sized areas. The separating fins possess a curvature to avoid preferred fluid movement. Within each area, hexagonal arranged pins with a distance of 1.2 mm to each other are arranged for better heat flux and catalyst bed stabilization. In the cavity of this structure, catalyst particles of a size 200 to 300 µm are distributed compactly to form a catalyst bed. Along the reactor length, in this case the radius, the flow cross-section is continuously increasing. The reason for this is a reduction in residence time due to the extreme formation of gas, which can be partly or completely compensated by this shaping depending on the degree of dehydrogenation (DoDH). Figure 2b shows the reactor with an integrated microstructure. The loaded LOHC is fed from below and then enters the microstructure. The radially removed product mixture is collected and transported to one outlet on the side. A steel plate above the microstructure can be replaced by a window, which makes an optical inspection of the processes in the catalyst bed possible. The reactor is electrically heated via heating cartridges and sealed between the individual components by flat graphite gaskets.

**Figure 3:**
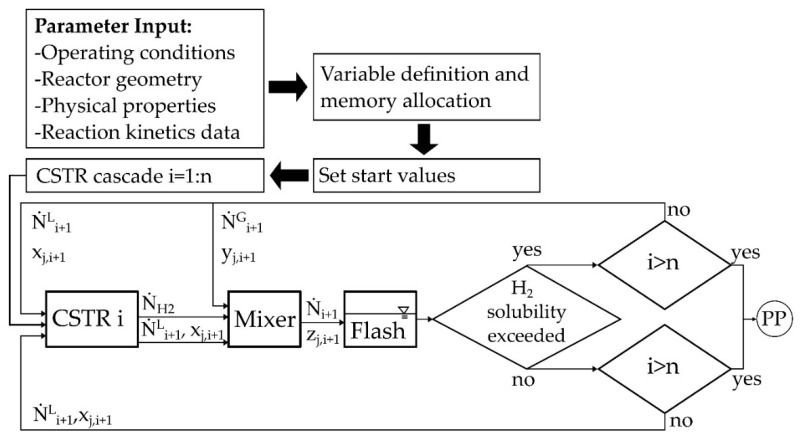
Program flow chart of the model for the radial flow reactor. In this Tanks-in-series model, the phase equilibrium and physical absorption is calculated at every single tank.

In the reactor model, it is assumed that the flow is approximately similar to that of a Plug Flow Reactor (PFR). Thus, a 1-dimensional problem, referred to the radius as coordinate, is assumed. A Tanks-in-Series model, which represents fluid-connected cylindrical rings as segments of the reactor along the radius coordinate with at least 50 tanks, is applied for the description of the PFR. A program flow chart can be seen in Figure 3. After defining the input parameters and setting the start values, the program calculates the CSTR (Continuous stirred tank reactor) cascade. In each tank (CSTR), the molar flows of the gas phase ${\dot{N}}_{H2}$ and liquid phase ${\dot{N}}^{L}$ as well as the molar fractions $x_{i}$ are determined. In a mixer, gas and liquid phase are summed up to molar fractions of $z_{i}$ and a flashbox is applied to calculate a new gas-liquid distribution of all species for the next tank based on an ideal gas law or with the modified UNIFAC model. Subsequently, it is checked whether the liquid phase is already saturated with Hydrogen. If not especially at the inlet of the reactor, the generated hydrogen is assumed to absorb until the saturation is exceeded and the free gas phase is formed. Physisorption of hydrogen in the LOHC was taken into account by Henry’s law. In addition to the reactor geometry, necessary substance data were also implemented [17,18,19,20]. The modelling was performed with the software Matlab® and the equations were solved with the solver “fsolve”.

The formation of hydrogen takes place under consecutive forming of double bonds at the catalyst surface. Due to the unfavorable energetic conditions of many possible intermediates, only the hydrogenation stages with fully hydrogenated/dehydrogenated C6-rings can experimentally be determined as species (12H-DBT, 6H-DBT) [21]. In the DBT reaction network, there are, thus, four species considered, each with a large number of isomers. Due to the complexity of the reaction kinetics, adsorption and desorption phenomena were neglected. Since a catalyst in egg-shell configuration is used, only an external mass transport limitation can exist. Furthermore, due to the limited availability of isomer data, it was further assumed that the isomers of the respective LOHC species are chemically and physically identical. This results in three equilibrium reactions shown below.

(1) $$18H-DBT\;\overset{k_{1}}{\underset{k_{4}}{⇄}}\;12H-DBT+3H_{2}$$

(2) $$12H-DBT\;\overset{k_{2}}{\underset{k_{5}}{⇄}}\;6H-DBT+3H_{2}$$

(3) $$6H-DBT\;\overset{k_{3}}{\underset{k_{6}}{⇄}}\;0H-DBT+3H_{2}$$

The experiments were carried out under conditions where hydrogenation can be neglected compared to dehydrogenation, i.e., at low conversion and far away from thermodynamic equilibrium.

(4) $$k_{1}\gg k_{4},\;\;k_{2}\gg k_{5},\;\;\;k_{3}\gg k_{6}$$

For the experiments, an almost complete hydrogenated perhydro-dibenzyltoluene (hydrogenation degree 96.1%, provided by Hydrogenious Technologies GmbH) was used as starting material. This means that there is nearly exclusively 18H-DBT in the feedstock, which is why only the first reaction was considered in the experiments (see Equation (1)). It was also taken care in the experiments that 6H-DBT and 0H-DBT were below the quantification limit. Nevertheless, the determined reaction constant *k*_1_ was used as the reaction constant of the other reactions in the model, i.e., the further simulation.

(5) $$k_{1}=k_{2}=k_{3}=k$$

To keep the conversion low, the catalyst bed was diluted with inert material to 25 wt%.

In summary of the kinetic assumptions including further a reaction order of *n* = 1, the following simple rate expression results for all reaction steps.

(6) $$r_{i}=k\cdot c_{i}^{n}\;\mathrm{with}\;c_{i}=\frac{x_{i,out}\cdot \rho_{LOHC,mix}^{liquid}}{{\tilde{M}}_{LOHC,mix}}$$

The reaction constant is described by the modified Arrhenius equation by applying a reference temperature.

(7) $$k=k_{T,ref}\cdot \exp\left(\frac{-E_{A,R}}{R}\cdot \left(\frac{1}{T}-\frac{1}{T_{ref}}\right)\right)$$

A total number of six reaction temperatures were experimentally investigated with each having four different residence times, so that a total of 24 measured data points were generated. The degree of dehydrogenation was determined by NMR spectroscopy [21]. A micro-ring gear pump was used to adjust the modified mass-related residence time, according to the following equation by applying the initial LOHC feed.

(8) $$\tau_{mod}=\frac{m_{cat}}{{\dot{m}}_{LOHC}}$$

### 2.2. Membrane Apparatus

**Figure 4:**
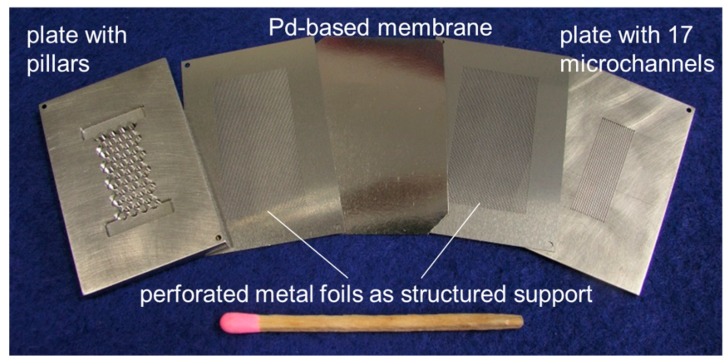
Picture of the used microstructured membrane separation module [16]. Due to low operating temperatures (300–350 °C), a PdAg membrane provided by SINTEF/Oslo was selected.

A membrane module with 17 microchannels (width × depth × length: 500 µm × 300 µm × 4 cm), which was described in detail in References [9,16], was used as separation device (see Figure 4). The used PdAg membrane was produced by SINTEF/Oslo via magnetron sputtering and has a thickness of approximately 10 µm. The membranes are produced by deposition on the perfect surface of 6 inch silicon wafers from an alloyed Pd77Ag23 target. Subsequently, the film was removed from the wafer, which allowed for the integration in a module [22].

In general, PdAg is more suitable for the LOHC application than pure palladium due to lower operating temperatures (300–350 °C) and higher hydrogen permeation flux, which fit to the dehydrogenation operating conditions. Microsieves from both sides were used to mechanically stabilize the membrane. Based on the number of holes in the sieve, an effective membrane area of 1.5 cm² was calculated. Good stability of these membranes has been reported several times in various studies. However, long-term operation of these membranes under the load with DBT has not been experimentally verified yet. This part will follow in further studies.

The separation of hydrogen via Pd-based membranes is a multi-step mechanism. If the limiting step is bulk diffusion in the Pd lattice, the flux can be described by combining Fick’s and Sieverts’ law with a square root dependence on the hydrogen pressure.

(9) $${\dot{F}}_{H_{2}}=\Pi \cdot \left(p_{H_{2},Ret}^{0.5}-p_{H_{2},Perm}^{0.5}\right)$$

Thus, the Flux is dependent on the permeance and the square root of the partial pressure gradient of hydrogen between the permeate side and the retentate side. The permeance *Π* is defined as the quotient of temperature-dependent and material specific permeability *Q* and membrane thickness *s*. The temperature-dependency follows the Arrhenius expression below.

(10) $$\Pi =\frac{Q}{s}=\frac{Q_{0}\cdot \exp\left(-\frac{E_{A,M}}{RT}\right)}{s}$$

The model reported in Reference [9] was used for fitting the experimental data and was used to determine the activation energy and the pre-exponential factor. Experiments were conducted at ambient hydrogen pressure on the permeate side (no sweep gas). To determine the permeance of the PdAg-membrane, a temperature and pressure variation at a constant hydrogen flow of 250 mL/min was carried out.

### 2.3. Multi-Stage Reactor Concept with Intermediate Hydrogen Separation

**Figure 5:**
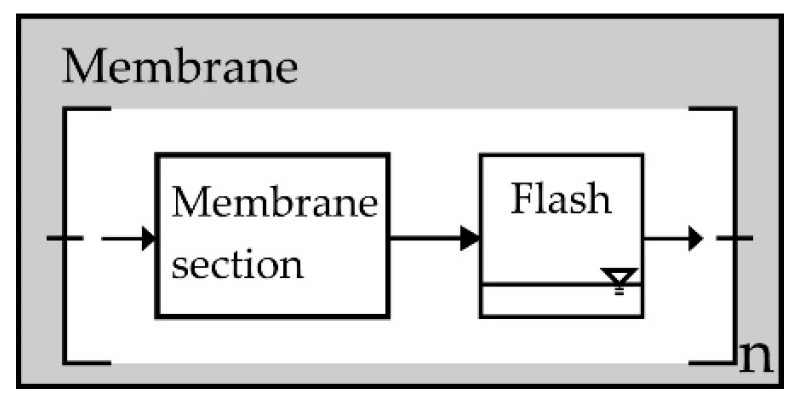
Modeling of the separation stage. The device was split into n parts to integrate a flash that takes into account the condensation of the LOHC.

For describing the sequence of devices, both mathematical models were linked according to the connected fluid streams. Figure 1 shows an example of a three-stage process. The gas and liquid phases are separated at the reactor outlet. The liquid product flow of stage *n* is fed directly into the following reactor stage *n* + 1. The gas phase, on the other hand, only enters the membrane separation module where it is separated from LOHC species in the gas phase. As a consequence, LOHC in the gas phase condenses on the membrane during the separation and the associated partial pressure reduction of the hydrogen. The condensed flow is also fed to the next reactor stage. To consider condensation for volume contraction and partial pressure change, the membrane was divided into *n* sections and a flash calculation (as described for the reactor simulation—ideal or by modified UNIFAC model) was integrated, which is shown schematically in Figure 5.

## 3. Results and Discussion

In this section, the experimental results are first discussed and then the multi-staged reactor concept with intermediate separation of hydrogen is evaluated.

### 3.1. Determination of the Reaction Kinetics

**Figure 6:**
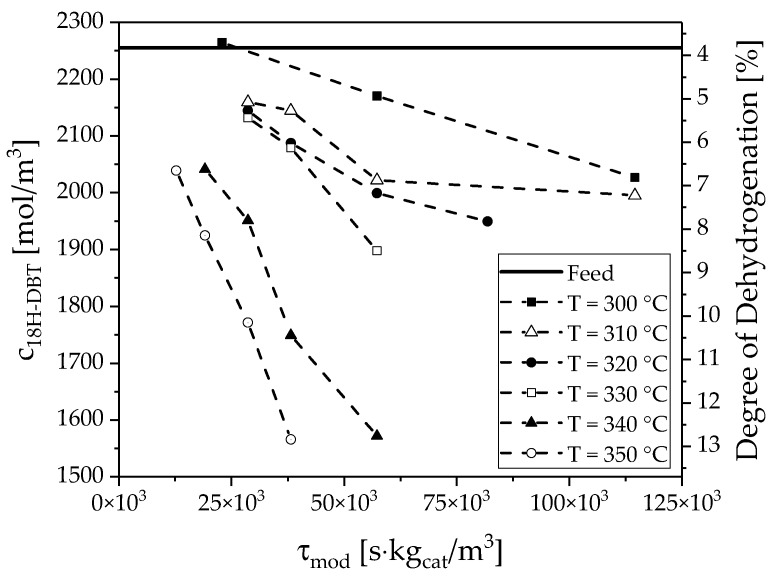
Concentration of 18H-DBT as a function of residence time at six different temperatures (300–350 °C) and 4 bar(a).

Figure 6 shows the concentration curves as a function of the modified residence time. An almost linear dependence can be seen for all temperatures. This is in agreement with expectations and shows that the residence time of the liquid at low conversions is close to the hydrodynamic residence time. The solid line indicates the degree of dehydrogenation of the feed stream due to incompleteness of hydrogenation. Dehydrogenation degrees between 1% and 9% were achieved in the experiments, which are low enough to be analyzed in a differential approach.

**Figure 7:**
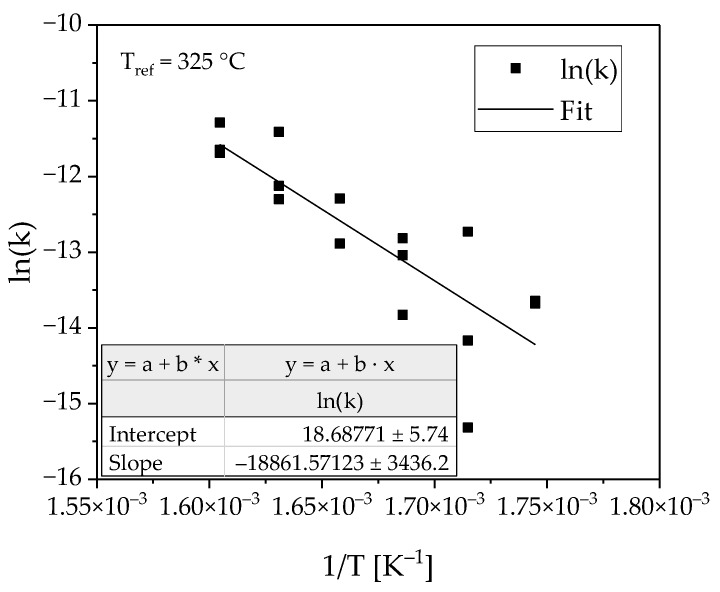
Arrhenius plot with average reaction constants and linear fit.

To determine the temperature dependency of the reaction, the logarithmic reaction rate was plotted as a function of temperature (see Figure 7). The linear fit describes the measuring points with satisfying quality, considering the complexity of the multiphase reaction system.

The following parameters could be determined from the fit.

(11) $$E_{A,R}=156.8\pm 28.5\;\;\mathrm{kJ}/\mathrm{mol}$$

(12) $$k_{T,ref}=2.637\times 10^{-6}\pm 0.307\times 10^{-6}\;\;m^{3}/\left({\mathrm{kg}}_{\mathrm{Cat}}s\right)$$

The model parameter can be compared to a study from the researchers at Erlangen [10]. They operated a batch reactor to determine the kinetics of H18-DBT on the Pt catalyst similar to ours. Their parameters were obtained from experiments with higher conversions. This allows determining a reaction order, which was calculated in the range of 2. However, this result is then already influenced by the back reactions as well as possible transfer-hydrogenations occurring between the species. Thus, their reported activation energy and the pre-exponential factor consequently differ. A value of $E_{A,R}$ of roughly 120 kJ/mol is calculated. Nevertheless, while higher conversions would be required to investigate the actual wetting of the catalyst (see Section 3.3), back-reactions and transfer-hydrogenations are influencing the results. More experiments will follow where a second or third stage entry concentration will be fed to the reactor to detail the kinetics further.

### 3.2. Membrane Characterization

**Figure 8:**
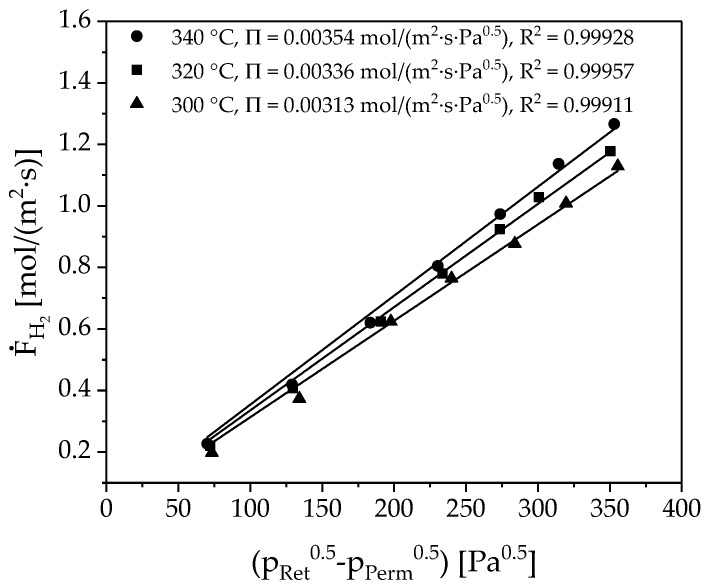
Sieverts plot of the permeation experiments. The permeance was determined for three temperatures by varying retentate pressure at an ambient hydrogen pressure at the permeate side.

The measured flux in the membrane device is plotted as a function of the difference between the square roots of the hydrogen partial pressures (see Figure 8). The measuring points at constant temperature follow the expected linear dependence. The Sieverts’ law can, therefore, describe permeation. The slope of each trend line represents the permeance.

**Figure 9:**
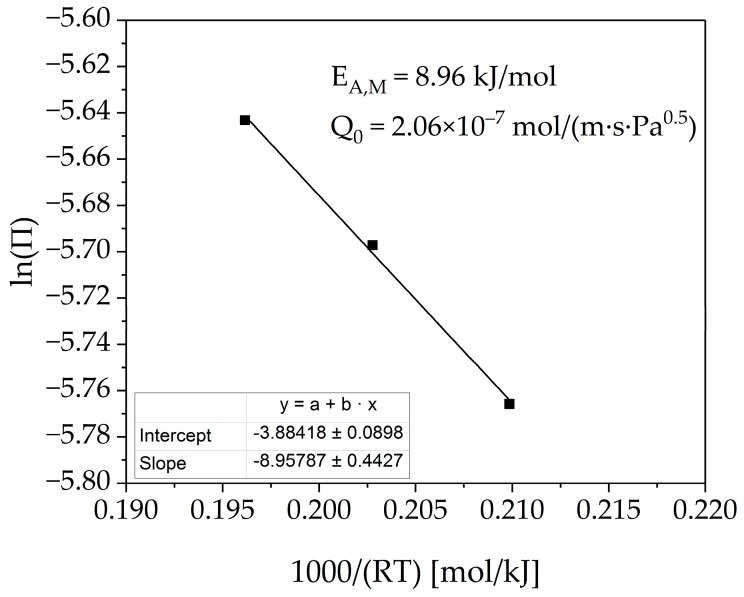
Fitted Arrhenius-relation of the measured permeance at three different temperatures.

If the logarithmus naturalis of permeance is plotted as a function of the reciprocal temperature, as seen in Figure 9, the activation energy and the pre-exponential factor can also be determined. The fit describes the measurement data with satisfying quality.

The following experimentally determined values were obtained, which seem to align to our previous studies [9] including the literature review.

(13) $$E_{A,M}=8.96\pm 0.44\;\;\mathrm{kJ}/\mathrm{mol}$$

(14) $$Q_{0}=2.06\times 10^{-7}\pm 0.05\times 10^{-7}\;\mathrm{mol}/\left({m\cdot s\cdot \;\mathrm{Pa}}^{0.5}\right)$$

### 3.3. Evaluation of the Multi-Stage Reactor Approach with Intermediate Hydrogen Separation

Based on the experimentally determined correlations, the results of the simulations with the multi-stage reactor concept with intermediate separation of hydrogen are presented in the following. To describe the unknown influence of the gas phase on the residence time of the liquid and the wetting of the catalyst a functional correlation between the effectively wetted catalyst mass and the real residence time of the liquid phase was introduced via the correction factor α. For this correction factor, a function was chosen, which correlates α to the void fraction of the liquid phase and the quotient of liquid and gas residence time as an exponent of the void fraction.

(15) $$m_{Cat}^{eff}=\alpha \cdot m_{Cat}$$

(16) $$\alpha =\ln\left(1+\varepsilon_{L}^{b}\cdot \left(e-1\right)\right)$$

(17) $$b=\frac{\tau_{gas}}{\tau_{liq}}$$

**Figure 10:**
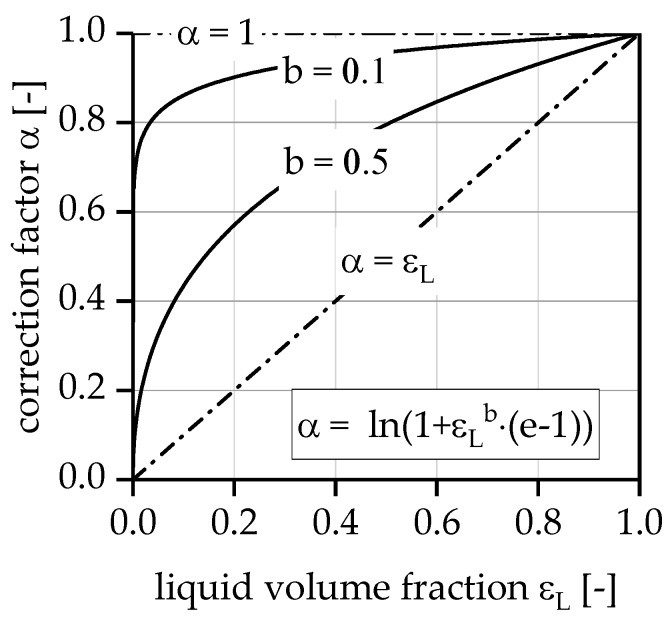
Constructed relationship between correction factor α and liquid void fraction $\varepsilon_{L}$ and the residence time proportion *b*.

As conversion increases, the liquid phase volume fraction $\varepsilon_{L}$ decreases and, finally, the effectively wetted catalyst mass that can serve the active surface for dehydrogenation of the liquid species decreases (see Figure 10). The chosen function allows us to describe the limiting cases. If gas and liquid velocity are identical, the void fraction and α have an almost linear dependence $\alpha \approx \varepsilon_{L}$ since $\tau_{gas}\approx \tau_{liq}$ or *b* = 1. According to our first observations in the packed bed microreactor system, it seems that part of the evolving gas phase can escape underneath the glass plate at much higher velocity and $\varepsilon_{L}$ can be greater than the expected value, which means that $\tau_{gas}\to 0$ i.e., high superficial velocity of the gas. This results in a much better case of α=1. All intermediate conditions where $\tau_{gas}<\tau_{liq}$ can be controlled by using parameter b, the residence time proportion (IRTP) is shown in the figure below; calculation according Equation (17).

**Figure 11:**
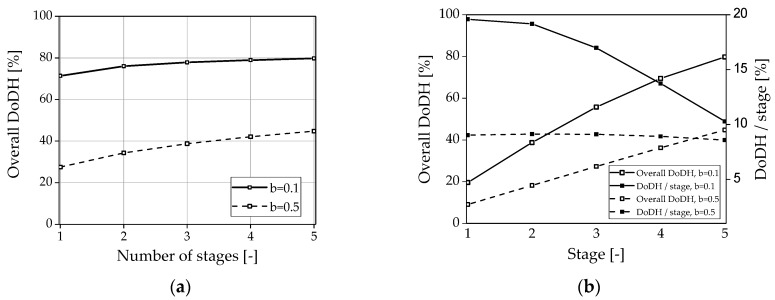
Simulation results (n_CSTR_ = 100) of the multi-staged approach with intermediate hydrogen separation carried out at 335 °C and 20 g/h feed: (**a**) overall DoDH with varying stage numbers at the same overall catalyst mass for two different parameters *b*. (**b**) Overall DoDH and DoDH per stage for a five-stage reactor concept.

Based on these assumptions and the experimentally determined data, simulations were performed based on the geometry of the investigated lab scale single-foil reactor system. The results can be seen in Figure 11 for a fixed reaction temperature and feed flow. First, it was investigated how the number of separation stages affects the overall degree of dehydrogenation (DoDH) with varying distribution of gas and liquid phases at a constant total catalyst mass in the reactor arrangement (see Figure 11a). That means that a single reactor is compared to two, three, four, and five reactors with half, third, fourth, or fifth mass in each reactor, respectively. It can be seen that the DoDH can be increased slightly by the increasing number of intermediate separations (*b* = 0.1) if the residence time of the gas phase is short while good catalyst wetting is the consequence. Under these conditions, the membrane application is less valuable, i.e., does not provide reasonable advantage with regard to costs and system size. This could probably be improved by lowering the total catalyst mass i.e., when a lower total conversion is obtained. Nevertheless, the longer the gas remains or the less catalyst is wetted due to similar residence time of gas and liquid, the more the process can be intensified (*b* = 0.5) via the stepwise hydrogen separation. The DoDH can be practically doubled with a five-stage process. If the DoDH is monitored over this five-stage process (see Figure 11b), it becomes clear that the DoDH increases strongly independent of the residence time distribution between the gas and the liquid phase in both cases. With more efficient dehydrogenation (*b* = 0.1), however, the DoDH per stage (DoDH/stage) decreases after the first stage while it remains almost constant at *b* = 0.5. This can be explained by the inhibition of the reaction by the resulting completely dehydrated dibenzyltoluene.

One major issue that needs to be resolved in the overall model is the possible reaction of gaseous LOHC species on the dry region of the catalyst. This must be investigated in the future by separate gas phase reaction experiments and inclusion in the overall model. Nevertheless, we believe that the contribution will be small compared due to the low partial pressure of LOHC species in the gas phase and compared to the surface coverage with liquid species. Thus, process intensification by the multi-stage system will definitely remain dominant.

We further plan to extend the kinetics with the back reaction in the future. This is relevant when the overall conversion increases towards thermodynamic equilibrium due to transfer hydrogenation happening between the different hydrogenated intermediates of dibenzyltoluene. Under those conditions, the reaction rate can drop quite considerably. Such effect would further argue in the direction of even higher process intensification by the suggested multi-stage concept, i.e., when technical and economical relevant conversion is desired. Therefore, differential conversion with the first stage, which is a partially dehydrogenated product, are required to build up an even more complex kinetic model. Lastly, it is planned to perform optimization simulations to larger scale dehydrogenation systems based on the more detailed modeling.

## 4. Conclusions

In this work, a microstructured multi-stage reactor concept with intermediate separation of hydrogen for the purpose of dehydrogenation of perhydro-dibenzyltoluene was investigated. First, the kinetics for the reaction and the separation of the hydrogen were determined. The experimental data were used to feed a developed model describing the multi-stage approach. Simulations were carried out, which show that the described approach can drastically intensify the whole dehydrogenation process in addition to the purification of the hydrogen especially under conditions where gas has no superficial velocity. PdAg membranes are particularly suitable for use in this context due to their relatively high permeance at a low operation temperature (300–350 °C). Back reaction and gas phase reactions will be included in the model in future work to describe the promising effects toward process intensification of the intermediate hydrogen separation. Moreover, long-term testing of a lab model will further deliver data on stability, which will then be used to scale a plant for larger throughput and to perform life cycle analysis (costing and environmental suitability) of the proposed dehydrogenation system.
